# Detection of Unilateral Arm Paresis after Stroke by Wearable Accelerometers and Machine Learning

**DOI:** 10.3390/s21237784

**Published:** 2021-11-23

**Authors:** Johan Wasselius, Eric Lyckegård Finn, Emma Persson, Petter Ericson, Christina Brogårdh, Arne G. Lindgren, Teresa Ullberg, Kalle Åström

**Affiliations:** 1 Department of Medical Imaging and Physiology, Skåne University Hospital, 22185 Lund, Sweden; Teresa.ullberg@med.lu.se; 2 Department of Clinical Sciences, Lund University, 22185 Lund, Sweden; arne.g.lindgren@skane.se; 3 Uman Sense AB, 22381 Lund, Sweden; eric.finn@umansense.com (E.L.F.); petter.ericson@umansense.com (P.E.); 4 Centre for Mathematical Sciences, Lund University, P.O. Box 118, 22100 Lund, Sweden; tfy15epe@student.lu.se (E.P.); karl.astrom@math.lth.se (K.Å.); 5 Department of Neurology, Rehabilitation Medicine, Memory and Geriatrics, Skåne University Hospital, 22185 Lund, Sweden; christina.brogardh@med.lu.se; 6 Department of Health Sciences, Lund University, 22185 Lund, Sweden; 7 Department of Neurology, Skåne University Hospital, 22185 Lund, Sweden

**Keywords:** wearable, accelerometer, sensor, stroke, hemiparesis, motor deficit, monitoring, machine learning, artificial intelligence

## Abstract

Recent advances in stroke treatment have provided effective tools to successfully treat ischemic stroke, but still a majority of patients are not treated due to late arrival to hospital. With modern stroke treatment, earlier arrival would greatly improve the overall treatment results. This prospective study was performed to asses the capability of bilateral accelerometers worn in bracelets 24/7 to detect unilateral arm paralysis, a hallmark symptom of stroke, early enough to receive treatment. Classical machine learning algorithms as well as state-of-the-art deep neural networks were evaluated on detection times between 15 min and 120 min. Motion data were collected using triaxial accelerometer bracelets worn on both arms for 24 h. Eighty-four stroke patients with unilateral arm motor impairment and 101 healthy subjects participated in the study. Accelerometer data were divided into data windows of different lengths and analyzed using multiple machine learning algorithms. The results show that all algorithms performed well in separating the two groups early enough to be clinically relevant, based on wrist-worn accelerometers. The two evaluated deep learning models, fully convolutional network and InceptionTime, performed better than the classical machine learning models with an AUC score between 0.947–0.957 on 15 min data windows and up to 0.993–0.994 on 120 min data windows. Window lengths longer than 90 min only marginally improved performance. The difference in performance between the deep learning models and the classical models was statistically significant according to a non-parametric Friedman test followed by a post-hoc Nemenyi test. Introduction of wearable stroke detection devices may dramatically increase the portion of stroke patients eligible for revascularization and shorten the time to treatment. Since the treatment effect is highly time-dependent, early stroke detection may dramatically improve stroke outcomes.

## 1. Introduction

With the recent advances of machine learning and artificial intelligence algorithms, new frontiers are opening up within the field of medicine and as ambient support by sensors as described in recent reviews on Internet-of-Things- and ambient-assisted Living [1,2]. There are multiple examples of artificial intelligence aiding the physician in diagnosing illness or even performing some diagnostic tasks independently [3,4].

In the field of stroke care, artificial intelligence and wearable sensors have primarily been used to identify risk factors such as detection of atrial fibrillation using smartwatches [5,6]—reviewed in [7]—and blood pressure estimation based on photoplethysmography to assess hypertension [8]. Within stroke imaging, artificial intelligence has been used to perform several imaging processing tasks, reviewed in [9,10]. However, most of these applications are based on data gathered with a human interpreter in mind, such as the visual aspects of a skin lesion or the digital reconstruction of a chest X-ray.

Within the field of neurology, assessing patterns of movement is a vital part of the clinical evaluation of suspected neurological illness. This evaluation is typically based on the patient’s self-reported symptoms and on the physician’s clinical examination of the patient. The clinical evaluation, however, has few objective measure points and can only assess the patient’s current symptoms. Recently, several efforts have been done combining artificial intelligence and various sensors to detect the onset of stroke or to diagnose stroke or sub-types of stroke such as intracerebral hemorrhages, which is a field that was recently reviewed [11].

With the emergence of readily available, comfortable, and affordable wearable accelerometers, high-detail kinetic data can be continuously recorded with a low level of intrusiveness for the patient.

We hypothesized that wearable accelerometers combined with artificial intelligence could offer a novel method of continuously screening a patient for the development of motor symptoms, by real-time gathering and interpreting kinetic data. In the event of the patient suffering a motor deficit, the system could alert the patient and the patient’s next of kin to the incident, resulting in reduced patient delay in contacting health care services for further evaluation and potential treatment.

A stroke typically causes sudden unilateral motor deficit without any prodromal symptoms, which is present at onset in up to 83–90% of all acute stroke cases [12,13,14,15]. During the last decades, effective treatment for acute ischemic stroke has been developed [16,17,18]. However, the sudden onset and debilitating symptoms restrict the ability of patients to alert themselves upon onset, and patient delay is therefore a major limiting factor for successful treatment. In Sweden, only 17% of the 18000 patients with acute ischemic stroke registered in the Swedish Stroke Register during 2019 received reperfusion treatment, with the main limiting factor reported being late arrival to hospital [19]. Current time limits for reperfusion treatment in Sweden are 4.5 h for intravenous thrombolytic treatment and 6 h for endovascular thrombectomy. Although recent trials of MT 6–24 h after onset [20,21] have shown excellent results on a small portion of patients, it still generally remains true that ”Time is Brain” in the sense that minimizing the time from onset to treatment is essential for maximizing the treatment effect in the entire stroke population.

We believe that wearable accelerometers combined with signal processing and machine learning could offer a novel method of continuously screening a patient at risk for development of arm motor deficits. The concept of separating arms with motor deficit caused by stroke from healthy arms has been shown to work in several studies [22,23,24] and was summarized in recent systematic reviews [25,26].

### 1.1. Aims

We hypothesized that a system based on continuous analysis of wearable bilateral accelerometer signals can indicate a stroke by identifying the arm motor deficit within a short enough time from onset to be clinically relevant. This study aimed to analyze different machine learning algorithms’ ability to accurately separate stroke-affected arms from healthy arms in patients with remaining arm motor deficits from a recent stroke, compared to healthy volunteers.

### 1.2. Related Work

Several groups have studied bilateral accelerometry in stroke patients using experimental devices [22,23,27], performing predefined tasks typically involving both arms [28], or by accelerometer data truncated into defined “counts,” similar to step-counting devices [29] or based on analysis of time periods of several hours [22,23,24], which will work well for long-term surveillance of rehabilitation but not for detection of stroke onset in high-risk patients. Some previous work included only daytime registration [22,29], whereas Jeon et al. [30] have shown the ability to differentiate between healthy and diseased arms using accelerometry in stroke patients during sleep. “Wake-up stroke” presents a clinical challenge since the exact time of onset is unknown. The capability to identify stroke onset during sleep would be of particular clinical interest; therefore, we chose to register for 24 h to include time of sleep as well as time awake. The methodology used in this work was partly developed as part of a master’s thesis by two of the authors [31].

## 2. Materials and Methods

**Figure 1:**
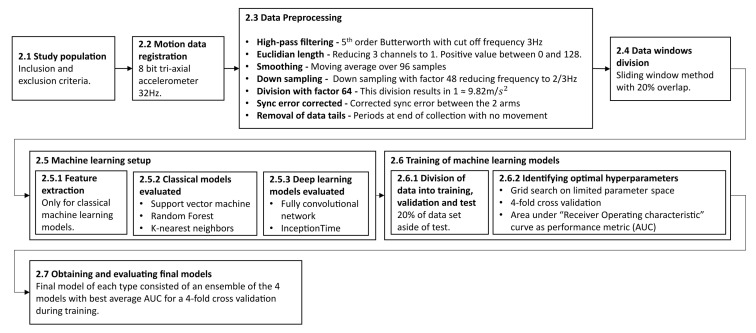
The outline of the methodology described in this study. It begins with the inclusion of patients and registration of data and describes the data preprocessing conducted, the machine learning setup and training, and finally the evaluation of the final models. Each step is described in more detail in the corresponding subsection.

The following section describes the methodology and experimental process in this work. The subsections are ordered in a chronological order to when they were performed. An overview of the entire process is outlined in Figure 1.

### 2.1. Study Population

Patients with a recent stroke were screened at a University Hospital stroke ward. Inclusion criteria were:

- Recent stroke with unilateral arm motor deficit; AND
- No previous condition affecting the arm motor function of the unaffected arm.

Exclusion criteria were:

- Age younger than 18; OR
- Inability to give informed consent; OR
- Unwillingness to participate.

Patient data, including NIHSS at admission, stroke type, demographic data, and pre-stroke modified Rankin scale (mRS), were obtained from the electronic patient record after consent (patient characteristics are seen in Section 3.1). The motor impairment was also subjectively graded in accordance with the NIHSS scale (NIHSS item 5; 0–4). All grades of motor impairment (1–4) were accepted into the study. This differs to some of the previous studies that focused on patients with mild or moderate impairment [22,24].

Additionally, healthy age-matched subjects were included as the control population. Inclusion criteria for the control population were:

- No previous condition affecting the arm motor function of either arm.

Exclusion criteria were:

- Age younger than 18; OR
- Inability to give informed consent; OR
- Unwillingness to participate.

All healthy subjects completed a questionnaire including demographic data and previous medical conditions. The local ethical committee approved the study (#2015-287), and all study activities were conducted in accordance with the Declaration of Helsinki.

### 2.2. Motion Data Registration

Motion data were recorded for both patients and healthy subjects using a wearable triaxial accelerometer bracelet on each arm (E4, Empatica Inc., Cambridge, MA, USA). Accelerometer data were collected and downloaded from the bracelets using the manufacturer’s software (E4 manager, Empatica Inc., Cambridge, MA, USA). Downloaded data included date and time, triaxial accelerometer data sampled at 32 Hz, and other sensor data not used here (4 Hz electrodermal Activity sensor (EDA), 4 Hz Infrared thermopile sensor, and 64 Hz photopletysmogram sensor (PPG). Data were further processed using Matlab (Mathworks, Cambridge, MA, USA).

### 2.3. Data Preprocessing

The triaxial accelerometers were sampled at 32 Hz. The measurements were 8 bits, i.e., integers in the range of −128 to 127, where the value 64 corresponded to $g\approx 9.8\;m/s^{2}$, which equates to the Earth’s gravitational effect on an arm in a resting state. Most of the time this value was close to 64 (in length), since the sensor was relatively stationary for long periods. We were primarily interested in deviations from this stationary state. In order to remove the effect of Earth’s gravity and ensure the robustness of the signal in each axis, we first performed a high-pass filter (fifth-order Butterworth filter with a cut-off frequency of 3 Hz) for each of the 3-axis channels independently. Then, the Euclidean length of the triaxial accelerometer measurements was calculated, resulting in a single value at 32 Hz. The resulting signal was low when there was no motion and high when there was motion, regardless of the direction of the motion. In order to further reduce the amount of data, we performed a low-pass smoothing filter (moving average over 96 samples) followed by subsampling by a factor of 48. The resulting signal thus corresponded to a 0.67 Hz sampling frequency. The signal values were then divided by a factor of 64 in order to normalize the data so that a value of 1 was equal to the Earth’s gravitational constant. This resulted in a a positive signal in the range 0–2, but with most values (99.92%) being below or equal to 1.

As the signal from each of the two arms had been collected independently using two separate wristbands, there were in some cases a small sync error between the two arms as a result of slightly different start-up times of the two bracelets. These sync errors were corrected by finding the optimal overlap where the absolute sum of the differences between the two arms at each measurement point was minimized.

The collected arm-movement data for each subject were then visually inspected in order to detect and remove additional data “tails” (recorded data at the end of series with no movement). These tails were a result of the bracelets not being immediately turned off at the end of recording session.

### 2.4. Dividing Data into Overlapping Windows Using a Sliding Window Approach

The collected streams of arm-movement data were divided into windows using a sliding window approach. This was done to ensure that the inputs to the different machine learning models would be of fixed length. This made it possible to utilize the same machine learning principles that are used for image classification as each window of data could be viewed as a narrow image two pixels wide and one window length long. Several different lengths of windows were evaluated to investigate the impact of window length on performance. The following window sizes were evaluated: 15 min, 30 min, 45 min, 60 min, 90 min, and 120 min. There is a trade-off between accuracy and speed determined by the window size [30]. A smaller window will allow for quicker detection but will also reduce performance. However, the window needs to be short enough in order for a detection to be clinically relevant. A 20% overlap between adjacent windows was utilized, which increased the amount of available data windows through augmentation.

### 2.5. Machine Learning Training Setup

Several different machine learning models were trained and evaluated to compare the performance between different algorithms. This included both traditional machine learning algorithms, relying on the extraction of features from the raw data as input, as well as deep learning architectures, which utilize raw data as input.

#### 2.5.1. Features Used for Classical Machine Learning

Before it was possible to train the classical machine learning models, it was necessary to extract features from the input data. When determining which features to include, we wanted to select robust features that we believed would capture the differences between the two arms well. We also wanted to include different features that were able to capture different characteristics in the two arms, both qualitatively like the mean and quantitatively like the number of occurrences of one arm being larger than the other. Some of the features considered were inspired from the ones used by Jeon et al. [30].

The selection of features was done by evaluating different feature combinations on a support vector machine with fixed hyperparameters to see which combination of features gave good results. Visual inspections of the two largest principal components was also conducted on the different feature combinations in order to evaluate how well the features separated the two classes.

Since the purpose of this study was to asses the level of classification performance possible to be obtained by comparing the different machine learning models, the most important aspect was that the same features were used on all evaluated classical machine learning models. We decided to use the following features from each window of data:

- Mean of arm 1 and arm 2.
- Median of arm 1 and arm 2.
- Standard deviation of arm 1 and arm 2.
- Max value for arm 1 and arm 2.
- Difference in mean between arm 1 and arm 2.
- Difference in median between arm 1 and arm 2.
- Difference in standard deviation between arm 1 and arm 2.
- Difference in max value between arm 1 and arm 2
- Number of occurrences arm 1 is at least 0.01 larger than arm 2 divided by window length.
- Number of occurrences arm 2 is at least 0.01 larger than arm 1 divided by window length.

It was partly due the difficult feature extraction and selection process when using classical machine learning models that deep learning models were also included in the evaluation. One of the main benefits with deep learning models is that is not necessary to conduct feature extraction as they are able to handle raw data as input, thus eliminating the need for feature extraction.

#### 2.5.2. Classical Models Evaluated

A total of three different classical machine learning classifier models were evaluated: support vector machine (SVM) with the radial basis function as kernel, K-nearest neighbors (KNN), and random forest (RF).

The SVM works by constructing a maximum margin separator, which is a separating hyperplane with maximum possible distance to the training points of the two classes [32]. This makes SVM a linear classifier, but by using a kernel, it can become possible for an SVM to separate non-linear data as well, since data which is not linearly separable in the original space may be linearly separable in a higher-dimensional space [32]. In this study, we used the radial basis function as a kernel [33]. In addition, as the data may not be perfectly separable with the radial basis function, we also relaxed the hard margin constraint to give a soft margin where some of the training points were allowed to be misclassified [33].

The RF consists of an ensemble of binary decision trees created by randomly varying the attribute choices, which makes RF a form of decision tree bagging [32]. Each decision tree is created by drawing a bootstrap sample from the training data. The following is applied to each node: a random subset of features is drawn from all available features, then the optimal variable split point is decided using appropriate splitting criterion, and the node is then split into two daughter nodes [34]. The process is repeated until the number of samples in a node is less than the decided minimum sample split [34].

The KNN works by classifying a new sample with the same class as the majority of the K closest samples in the training data; however, it is possible to apply other thresholds then the majority or 50% [32]. There are different distance metrics that can be utilized for KNN such as the Manhattan distance or the Euclidean distance. In this study, we used the Euclidean distance.

The three models were chosen as they may be used for classification and are capable of handling non-linear data. They were also used by Jeon et al. [30], where they performed well on a similar setting. It was decided not to use any linear models as the linear discriminant analysis (LDA) classifier from [30] performed considerably worse than the other evaluated classifiers. Therefore, it is likely better to use non-linear classifiers. In [30], they also evaluated the decision tree classifier. However, as RF is an ensemble of decision trees, it was decided to be enough to only include RF in this evaluation.

The Scikit-learn (version 0.24.2) implementation of the three classifiers were used in this study [35].

#### 2.5.3. Deep Learning Models Evaluated

In addition to the classical machine learning models, state-of-the art deep learning models were also included as part of the evaluation to see how well they would perform. This is something that was not part of the study by Jeon et al. [30]. In recent years, deep learning models have been increasingly used and have reached state-of-the-art performance on many of the available benchmark datasets in both image and time series classification [36]. Deep learning models have a high degree of complexity that makes it possible for them to adapt to very complex problems. Deep learning models are made up of layers of nodes that are connected through weights, typically an input layer, and several hidden layers followed by an output layer. When training a deep learning model, the goal is to optimize the value of the weights based on the training data and corresponding labels [32]. A major advantage with deep learning models is that it is possible to train deep learning models on raw input data, thus removing the need to manually extract relevant features. Instead, they are able to identify features from the raw input as part of the training [36].

The challenge with deep learning networks is to come up with a good network architecture. Therefore, it was decided to, instead of trying to design a new architecture for this specific problem, evaluate two state-of-the-art deep learning architectures for time series classification.

The first state-of-the-art network evaluated was the fully convolutional network (FCN) proposed by Wang et al. [37] and was shown to reach state-of-the-art performance on the UCR time series archive, which is one of the largest benchmark libraries for time series classification [38,39]. The fully convolutional network draws inspiration from AlexNet, which has reached good performance on image recognition tasks [37,40]. It consists of convolutional layers, which are broadly used in image classification problems, together with occasional residual connection, which helps in reducing the risk of the vanishing gradient problem.For complete description of this network, please see [37]. The TensorFlow implementation of FCN available in the companion repository to [38] was used in this study (https://github.com/hfawaz/dl-4-tsc/, accessed on 14 September 2021).

The second state-of-the-art deep neural network on time series classification that was evaluated in this study was InceptionTime, which has also reached state-of-the-art performance on the UCR dataset [41]. InceptionTime draws its inspiration from the Inception-v4 network designed for image classification and is made of custom blocks together with residual connections [42]. For a complete description of this network, please see [41]. The original InceptionTime model consists of an ensemble of five InceptionTime networks, but due to computational limitations, only one instance of the InceptionTime was used as the entire InceptionTime network in this study during training. The TensorFlow implementation of InceptionTime available in the companion repository to [41] was used in this study (https://github.com/hfawaz/InceptionTime, accessed on 14 September 2021).

In an effort to make training of the deep neural networks easier, especially on longer window lengths, an average pooling layer was added prior to feeding the input to the deep learning networks. The motivation was that networks trained using longer window lengths would not need the high resolution of data but that it instead would be more beneficial to train on lower resolution in order to better be able to take into account the entire window.

### 2.6. Training of Machine Learning Models

#### 2.6.1. Division of Data into Training, Validation, and Testing

The collected arm-movement data were divided into five groups, each containing about 20% of the subjects. The random split was made so that each subgroup had roughly the same class ratio as the entire dataset. One of the five datasets were labeled test and put aside to be used to evaluate the final trained models. The remaining datasets were labeled train-1 to train-4. These four datasets were used during development for training and validation using four-fold cross-validation.

#### 2.6.2. Identifying Optimal Hyperparameters

In order to identify a good selection of hyperparameters for each of the evaluated models, a grid search was conducted on a limited set of hyperparameters using a four-fold cross validation. For each combination of hyperparameters a total of four model instances was trained. Each model instance was trained by using one of the four training datasets as validation and the rest for training. As a result, each hyperparameter configuration was evaluated on all training data once. The average performance over the four iterations was used to evaluate a specific combination of hyperparameters. To reduce any potential bias caused by uneven prevalence between right- and left-sided strokes, all models were trained on both the original training data as well as a copy of the training where the two arms had been swapped.

The classic models KNN, SVM, and RF were all trained using the default stopping criteria implemented in Scikit-learn [35].

Due to the large flexibility of the deep learning models, and as a result a high risk for overtraining, a different stopping criteria was used. The deep learning models were all trained for 100 epochs. After each completed epoch, the current model state and validation accuracy on the validation data was saved. Upon completion of all 100 epochs, the model state resulting in the highest validation accuracy was chosen as the final trained model. A reduction in the learning rate was also used in order to improve training for the deep models. If no improvement was made over 20 epochs, the learning rate was reduced by 50%.

Different hyperparameters were included in the grid search for different types of machine learning models. For KNN classifiers, the “number of neighbors” taken into consideration when classifying were the only hyperparameter included in the grid search. For SVM, the hyperparameters’ “C-value,” a regularization parameter, and “γ-value,” a kernel coefficient, were included in the grid search. For RF, the hyperparameters’ “number of trees,” specifying the number of trees included in the random forest; “minimum sample split,” which is the minimum number of samples required for a branch to split into two leaves; and the split criteria, either “gini” or “entropy,” were included in the grid search. See the Scikit-learn documentation for a full explanation of the different hyperparameters [35].

For the deep learning models, FCN and InceptionTime, the initial learning rate and the downsampling factor were the hyperparameters used in the grid search. The learning rate controls how much the weights should be updated based on the error rate each time the weights are updated. The downsampling factor specified the size of the average pooling on the input data prior to providing it to the deep learning networks. As an example, a downsampling factor of 2 means that input data with the size 2 × 600 will be reduced to 2 × 300 using average pooling of each two adjacent samples.

#### 2.6.3. Evaluating Model Performance

Each model configuration was evaluated using the area under the curve (AUC) as a performance metric, which is the area under the receiver operating characteristics (ROC) curve. The ROC curve plots the the sensitivity against the specificity [43]. In order to calculate the AUC value, it is necessary to use a confidence score for a given prediction by a model rather than a binary prediction as it is different thresholds applied to the confidence score that produce the ROC curve.

Due to the differences in how the models classify a sample as either being positive or negative, different confidence scores were used. The proportion of trees with a positive classification score for a given input was used as a confidence score for random forest. The decision function, where the sign of the value determines the classification, was used as the confidence score for support vector machine, and the proportion of positive neighbors was used as the confidence score for K-nearest neighbors. For the deep learning networks, the value of the output node, which represented a positive classification, was used.

#### 2.6.4. Obtaining and Evaluating the Final Models

As the validation data had been used as an early stopping criteria during the training of the deep neural networks in order to avoid overtraining, it was not possible to retrain them using all available training data with the optimal hyperparameter configuration identified during the grid search. If all training data, equivalent to 80% of the total data had been used as part of training, there would be no data left to use for validation as part of the early stopping method for the deep models. Using the test data as validation would risk the final model becoming biased towards the test data. Therefore, it was decided to let the final model configuration consist of an ensemble of the four trained models during the four-fold cross validation, which resulted in the highest average AUC value. By using an ensemble of all four model instances, it was possible to make use of all available training data as all data would have been part of the training dataset for three out of the four models in the ensemble. Each trained ensemble was then evaluated on the test set, which was 20% of the total data that was set aside earlier, to see how well the models performed and generalized on never-before-seen data. The average confidence score of the four models was used as the resulting classification output.

In order to determine if the performance between different models was statistically significant, a non-parametric Friedman test followed by a post-hoc Nemenyi test was conducted as described in [44]. The non-parametric Friedman test was used to see if it was possible to discard the null hypothesis that all model performances were equally good, and the post-hoc Nemenyi test was used to pairwise analyze if the differences in model performance were statistically significant [44]. When conducting the statistical significance test, the test was divided into 10 smaller parts to simulate an evaluation of the models on multiple datasets to measure the consistency of their performance and to determine if their performance was statistically significant. The result of the statistical significance analysis was then summarized in a critical difference diagram [44].

## 3. Results

### 3.1. Study Population

Eighty-five (85) patients with unilateral arm motor impairment due to a recent stroke were included after giving written informed consent. One patient was wrongfully included (no remaining arm motor deficit at inclusion) and therefore excluded from the final analysis. One-hundred-two (102) healthy subjects were included in the control population. One healthy subject subject was wrongfully included (persistent arm motor deficit from a previous neurological condition that was not reported at inclusion) and was also excluded from the final analysis. In total, bilateral accelerometer data from 84 patients with arm motor deficit from a recent stroke and 101 healthy subjects were included in the final analysis.

Table 1 shows the study population characteristics from the patients and control individuals included in the study. The two individuals excluded from the final analysis are not included the table.

### 3.2. AUC Performance on Test Set

**Figure 2:**
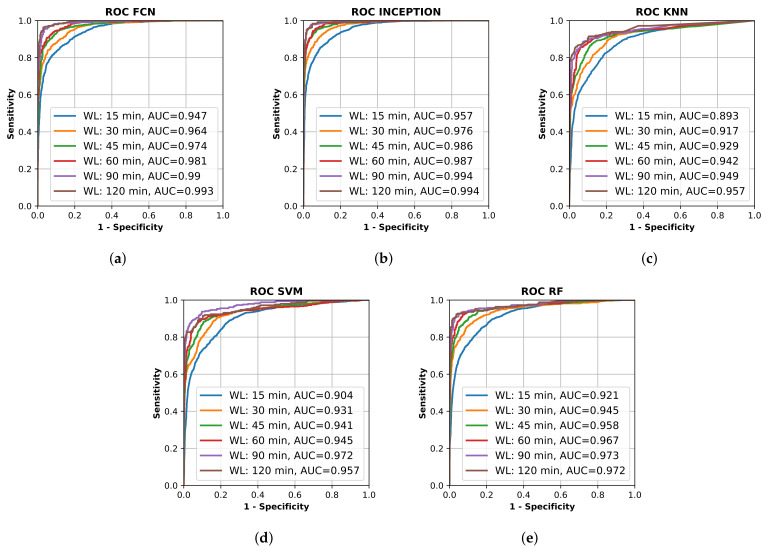
Receiver operating characteristic (ROC) curve for different trained machine learning models using different window lengths. (**a**) shows the deep learning classifier fully convolutional network (FCN); (**b**) shows the deep learning classifier InceptionTime (INCEPTION); (**c**) shows the K-nearest neighbors (KNN) classifier; (**d**) shows the support vector machine (SVM) classifier; and (**e**) shows the random forest (RF) classifier. Plots created using Matplotlib [45].

**Figure 3:**
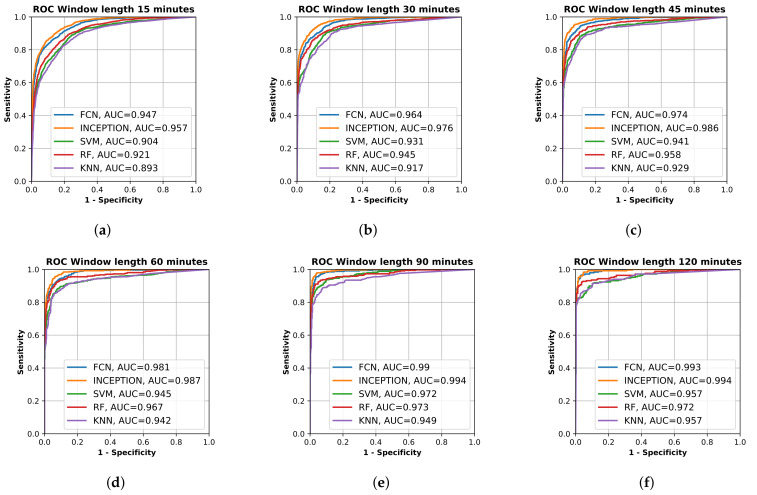
Receiver operating characteristic (ROC) curve of the trained models, fully convolutional network (FCN), InceptionTime (INCEPTION), support vector machine (SVM), random forest (RF), and K nearest neighbor (KF), on the test set grouped by input window length. (**a**) shows model results for 15 min window length; (**b**) shows model results for 30 min window length; (**c**) shows model results for 45 min window length; (**d**) shows model results for 60 min window length; (**e**) shows model results for 90 min window length; and (**f**) shows model results for 120 min window length. Plots created using Matplotlib [45].

All final models were evaluated on the test set, which was set aside early on during the data processing. Figure 2 shows the resulting ROC curves and AUC score for every evaluated classification model on each evaluated window length. Figure 3 shows the same curves as in Figure 2 but grouped by each evaluated window length instead of each evaluated model.

Figure 2 and Figure 3 show that the deep learning networks performed overall better than the classical machine learning models as they obtained a higher AUC on each evaluated window length. The deep learning models, FCN and InceptionTime (INCEPTION), had similar performance with INCEPTION obtaining slightly higher AUC scores compared to FCN.

**Figure 4:**
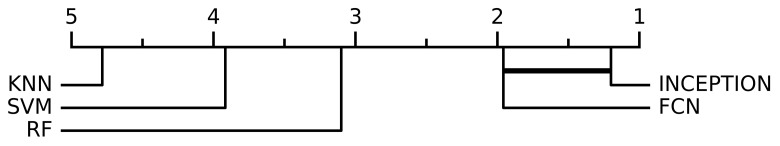
The critical difference diagram of the Nemenyi test with significance level α=0.05 for the different evaluated models. The test set were divided into 10 equally sized parts when performing the analysis, which combined with the 6 window lengths analyzed in this study (15 min, 30 min, 45 min, 60 min, 90 min, and 120 min) and resulted in a total of 60 evaluations for every model. The critical difference diagram shows that the FCN and INCEPTION models’ performance were not statistically significant according to the Nemenyi test. FCN and INCEPTION were, however, statistically significantly better compared with the other evaluated models, SVM, RF, and KNN, according to the test. The performances of SVM, RF, and KNN were also statistically significant different compared with each other.

When performing the non-parametric Friedman test, the null hypothesis that all model performances were equal could be rejected with a significance level of *p* < 0.0001. Figure 4 shows the resulting critical difference diagram as a result of the post-hoc Nemenyi test with the significance level of α=0.05, which was conducted after the null hypothesis was rejected. Figure 4 shows that all model performances were significantly different from each other except the two deep learning models, FCN and INCEPTION, which could not be separated from each other, but the Nemenyi test showed that booth the deep learning models were significantly better than all of the three classical models: SVM, KNN, or RF.

From Figure 2 and Figure 3, it is clear that the used input window length had the largest impact on performance compared with the model; however, the difference between window lengths decreased as the window length increased. There was only a minor overall difference in the AUC score for the different models between window lengths of 90 min and 120 min.

**Figure 5:**
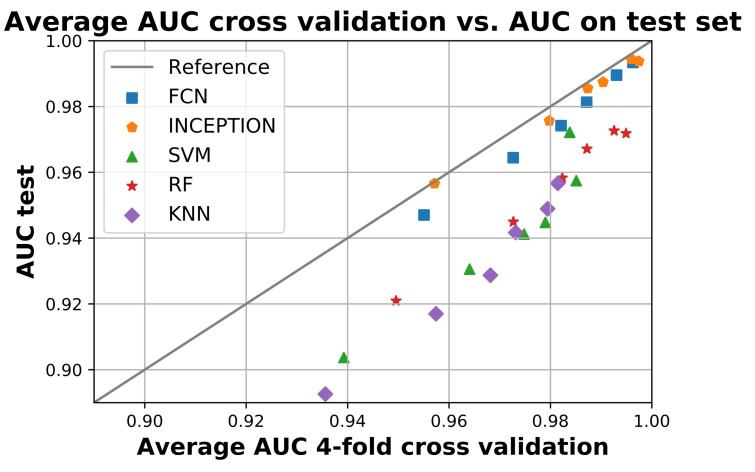
The figure shows the average area under the curve (AUC) during the 4-fold cross validation on the x-axis and the AUC when applied on the test set. All models below the reference line performed worse on the test set compared to training and vice versa. The figure shows that no model performed better on the test set compared to training, but the deep neural network model InceptionTime (INCEPTION) had the smallest overall difference. Figure created using Matplotlib [45].

Figure 5 compares the average AUC from the four-fold cross validation during training with the obtained AUC score when the models were applied to the test set. The figure shows that all models performed worse on the test set compared to training as they are all below the reference line. The deep learning models FCN and INCEPTION were overall closer to the reference line compared with the classical models. There was only a minor difference between the two deep learning models, with INCEPTION performing slightly better as it is overall closer to the reference line.

The hyperparameter values for the final models obtained from the grid search is presented in Supplementary Table S1.

In addition to the ROC curves and AUC values presented in Figure 2 and Figure 3, the sensitivity values, specificity values, and corresponding F1-score for the point on the ROC curve closest to [0, 1] are shown in Supplementary Table S2.

## 4. Discussion

This prospective study was performed in order to assess the capability to identify stroke-affected arms and healthy arms based on bilateral accelerometer data within a short-enough time to be clinically relevant. Unilateral paresis was chosen as a biomarker of stroke because it is a very common symptom present at the onset in as many as 83–90% of all acute stroke cases [12,13,14,15] and is relatively uncommon in other diseases.

### 4.1. Limitations

This study had several limitations. Firstly, the stroke-affected arms were all from patients with a recent stroke with persistent arm motor deficits, typically within a couple of days after onset, i.e., the onset of the stroke was not included in the accelerometer data. We think this is an acceptable model since the arm impairment is typically similar or less compared to the deficit at onset. Secondly, the accelerometer data from patients with a recent stroke was gathered in the hospital environment rather than in the home of the patient; therefore, activities during the hospitalization may affect the data. Thirdly, data were typically gathered from all included subjects for 24 h. Longer registration periods could potentially identify rare events not observed in our current dataset. Lastly, we used only one type of accelerometer device in this study.

The strengths and novel aspects of our work include: (1) the use of a commercially available accelerometer platform; (2) the inclusion of all grades of arm motor impairment (NIHSS sub-item 5; 1–4p), thereby ensuring that the detection ability is valid for all degrees of arm motor deficit, from mild impairment to complete arm paralysis; and (3) use of time-frames short enough to be clinically relevant for stroke onset detection.

### 4.2. Analysis of Results

The machine learning algorithms used in this study on accelerometer data from stroke patients with unilateral arm motor impairment and healthy subjects were able to discriminate between the two classes on time intervals shorter than those used in previous studies [22,23,24,29]. We also showed that it is possible to separate the two classes well throughout the 24 h collection period compared with previous studies that only focused on sleep periods [30]. The detection results, seen in Figure 2 and Figure 3, are in line with those achieved in a previous study that only focused on the night period [30].

The results show that all trained models were able to separate the two classes well. The sensitivity and specificity of detection of the stroke-affected arms increased with the window length used for analysis (Figure 2), which would be expected. However, between a 90-min window length and a 120-min window length (Figure 3e,f), there was only a minor improvement in performance for most evaluated models, suggesting that there is not much to gain by increasing the window length beyond 90 min. The support vector machine (SVM) even showed a decrease in performance between 90 and 120 min, which could be a result of fewer windows to train on due to the increased window length and fixed 20% overlap between adjacent windows.

The high AUC of 0.893–0.947 for windows as short as 15 min, and between 0.942–0.987 for windows of 60 min (Figure 3), in this population of mixed grades of motor impairment were surprising and encouraging, suggesting that accelerometry may be able to identify stroke onset within a short-enough time to allow medical treatment [46].

Out of the evaluated models, the deep neural network models FCN and INCEPTION obtained a significantly higher AUC score on all window lengths evaluated compared with the classical models (Figure 3). However, as seen in the critical difference diagram of the post-hoc Nemenyi test, shown in Figure 4, the difference in performance between FCN and INCPETION was not statistically significant, even though INCEPTION obtained a slightly better AUC score on the full test set for all window lengths evaluated (see Figure 3). Additional tests of the two models on new data may eventually determine if any of them is superior.

The trained models also generalized well according to the results of Figure 5. There was only a slight decrease in performance between the train and the test for all trained models. The decrease in performance on the test set was also similar for the same models regardless of window length, suggesting that the decrease may be due to the test set being slightly more difficult compared with the train set. If a particular model instance would have overtrained, then its performance would likely have been significantly worse compared with the others. Overall, the deep neural network models FCN and INCEPTION generalized the best as their performance was most similar between training and testing, where INCEPTION performed slightly better than FCN.

As there were, to our knowledge, no other studies on unilateral arm paresis collected from wrist-worn accelerometers worn for a continuous 24 h, it is difficult to do a performance comparison with other existing works. Jeon et al. [30] are the most similar, except that they only analyzed accelerometer data from sleep and used accuracy as their performance metric, which makes it difficult to compare with the AUC. However, when the sensitivity and specificity are equal, they will be same as the accuracy for that specific threshold. This makes it possible to estimate if the results of this study are similar to those obtained by Jeon et al. [30].

By looking at the point in the AUC graphs in Figure 3 where the sensitivity and specificity are equal, it seems that the results in this study are similar to those obtained by Jeon et al. [30]. The deep learning models evaluated in this study seem to reach slightly higher performance compared to those in Jeon et al. [30]. By looking at Figure 3a, the accuracy of the deep learning models on window length 15 min with equal sensitivity and specificity seemed to be about 85%, which is better than about 70% in [30], and from Figure 3b, the accuracy of the deep learning models on window length 30 min with equal sensitivity and specificity seemed to be about 90%, which is better than about 85% in [30]. The performance of the classifiers for detecting one-sided weakness also seemed to plateau around 70–80 min in Jeon et al. [30], which is similar to our results. The similarity in the results, when compared, supports the findings in this study.

The calculations needed for our analysis can easily be performed in real time. Evaluating one model on the entire test of 37 individuals, all with about 24 h of data, takes only a few seconds on a consumer-level personal computer. The time to evaluate the test set was similar for all models. The calculation of a single window will therefore take only a fraction of a second on a modern smartphone since modern smartphones have computing powers comparable to personal computers. Hence, it would be possible to conduct the calculations required in real time on a user’s smartphone as data is being recorded from wearable accelerometers, thereby providing several features required for a wearable stroke detection system or a stroke rehabilitation monitoring device.

Future research of interest on this subject would be to determine what level of sensitivity is possible to obtain if different restrictions are put on the number of false detections allowed during a specific time period. It would also be valuable to analyze what time of day the misclassifications occur, including if they are equally spread out or concentrated to some specific period of the day. In particular, it would be interesting to study the difference in performance between time awake and the time when a person is asleep.

## 5. Conclusions

In this work, we show that triaxial accelerometers worn in bilateral bracelets can identify stroke-affected arms with good performance. The two deep learning models evaluated performed significantly better compared to the classical machine learning models with an obtained AUC score between 0.947–0.957 for the 15 min window length and an AUC score between 0.993–0.994 for the 120 min window length. However, it was not possible to discern which of the two deep learning models were superior as their performance were similar. The evaluated models also generalized well between training and testing, suggesting that it will work well on new, never-before-seen data. The significance upon implementation of such wearable detection devices for use by patients with elevated risk of stroke would potentially shorten the time from onset to 911 dispatch and thereby all subsequent times within the stroke chain. This may dramatically increase the portion of stroke patients eligible for revascularization treatment and may shorten the time to revascularization, which directly affects patient outcomes—time is brain.

## Figures and Tables

**Table 1:** Study population demographics, including stroke sub-type and severity of the arm motor impairment.

Varible	Stroke Cases N = 84	Controls N = 101
Median age (range)	76.5 (35–98)	64 (22–92)
Female sex n (%)	30 (35.7)	53 (52.5)
Mean monitoring time, h (range)	24.8 (3.8–53.2)	25.5 (1.6–54.7)
Right side paresis n (%)	38 (45.2)	-
Grade of arm paresis (NIHSS item 5) n (%)		
No movement (4p)	19 (22.6)	-
No effort against gravity (3p)	36 (42.9)	-
Some effort against gravity (2p)	21 (25.0)	-
Drift (1p)	8 (9.5)	-
Pre-stroke mRS n (%)		
0	73 (86.9)	101 (100)
1	6 (7.1)	0
2	4 (4.7)	0
Unknown	1 (1.2)	0
Stroke subtype n (%)		
Large artery occlusion	34 (40.5)	-
Small artery occlusion	37 (44.0)	-
Intracerebral hemorrhage	12 (14.3)	-
Unknown	1 (1.2)	-
Acute treatment n (%)		
Intravenous thrombolysis (IVT)	9 (10.7)	-
Thrombectomy +IVT	12 (14.3)	-
No reperfusion treatment	62 (73.8)	-
Unknown	1 (1.2)	-

## Data Availability

Datasets of aggregated data can be provided upon reasonable request and after ethical committee approval.

## Supplementary Materials

The following are available online at https://www.mdpi.com/article/10.3390/s21237784/s1, Table S1: Identified hyperparameters for each model from the grid search, Table S2: Sensitivity, Specificity and F1-score for the different models corresponding to point in ROC graph closes to optimal [0, 1].
