# Supplementary material for: Detection of Unilateral Arm Paresis after Stroke by Wearable Accelerometers and Machine Learning
Source: Sensors (Basel). 2021 Nov 23;21(23):7784. doi: 10.3390/s21237784 (PMC8659933; doi:10.3390/s21237784)
Supplement: Supplementary file 1 [file sensors-21-07784-s001.zip › sensors-1453546-supplementary.pdf]

Supplementary material

Detection of Unilateral Arm Paresis After Stroke by Wearable Accelerometers and Machine Learning

Supplemental material 1 – Identified hyperparameters for each model from the grid search.

Supplemental Table 1 shows the identified hyperparameters found from the grid search for each of the five evaluated models "Fully Convolutional Network" FCN, InceptionTime (INCEPTION), Support Vector Machine (SVM), Random Forest (RF) and K-nearest neighbors (KNN) on the 6 different evaluated window lengths (15, 30, 45, 60, 90, and 120 minutes). The hyperparameters were identified through a grid search on a limited parameter space.

Supplemental Table S1

	Fully convolutional network		InceptionTime		Support vector machine		Random forest			K-nearest neighbors
**Window length**	**Learning rate**	**Down-sampling factor**	**Learning rate**	**Down-sampling factor**	**C-value**	**γ-value**	**Number of trees**	**Min sample split**	**Split criterion**	**Number of neighbors**
**15 min**	0.1	2	0.1	2	1	5	350	80	entropy	51
**30 min**	0.0001	4	0.0001	4	1	5	150	100	entropy	41
**45 min**	0.1	4	0.1	4	1	5	150	100	entropy	41
**60 min**	0.0001	8	0.0001	8	1	5	150	80	entropy	41
**90 min**	0.001	4	0.001	4	10	3	150	80	entropy	31
**120 min**	0.001	8	0.001	8	1	5	350	80	gini	17

Bye I'm gonna

Supplemental Table S1: The identified hyperparameters for the different classifiers from the grid search. Due to differences of the classifiers, different hyperparameters were included in the grid search for each classifier type. For the deep learning models, the hyperparameters were learning rate and down sampling factor. For the support vector machine, the hyperparameters were C-value and γ-value. For Random forest, the hyperparameters were number of trees, minimum sample split and split criterion. For the K-nearest neighbors classifiers the hyperparameter included were the number of neighbors.

Supplemental material 2 – Sensitivity, Specificity and F1-score for the different models corresponding to point in ROC graph closes to optimal [0, 1]

Supplemental Table 2 shows the sensitivity, specificity and calculated F1-score for each of the evaluated models on each evaluated window length using the threshold corresponding to the point the ROC diagram closest to [0,1]. Point [0, 1] represents optimal classification in a ROC diagram.

As seen in Supplemental Table 2 The model InceptionTime (INCEPTION) obtained the highest F1-score on the test set for most evaluated window lengths. For 120 minutes the Fully Convolutional Network (FCN) and InceptionTime (INCEPTION) obtained the same F1-score.

Supplemental Table S2

Window length	Model	Sensitivity	Specificity	F1-score	Window length	Model	Sensitivity	Specificity	F1-score
**15 min**	FCN	0.856	0.879	0.855	**60 min**	FCN	0.926	0.929	0.920
	INCEPTION	0.871	0.889	**0.869**		INCEPTION	0.943	0.950	**0.942**
	SVM	0.840	0.802	0.808		SVM	0.883	0.921	0.892
	RF	0.846	0.831	0.826		RF	0.922	0.929	0.918
	KNN	0.823	0.808	0.801		KNN	0.881	0.900	0.880
**30 min**	FCN	0.873	0.917	0.884	**90 min**	FCN	0.955	0.963	0.955
	INCEPTION	0.915	0.911	**0.905**		INCEPTION	0.973	0.963	**0.965**
	SVM	0.884	0.843	0.852		SVM	0.917	0.920	0.910
	RF	0.861	0.901	0.869		RF	0.926	0.949	0.931
	KNN	0.851	0.833	0.829		KNN	0.887	0.924	0.896
**45 min**	FCN	0.925	0.911	0.910	**120 min**	FCN	0.964	0.970	**0.964**
	INCEPTION	0.949	0.930	**0.934**		INCEPTION	0.960	0.974	**0.964**
	SVM	0.882	0.894	0.878		SVM	0.899	0.917	0.899
	RF	0.909	0.896	0.893		RF	0.927	0.957	0.937
	KNN	0.875	0.878	0.865		KNN	0.915	0.894	0.895

Supplemental Table S2: Sensitivity, specificity and calculated F1 score for each of the evaluated models on each evaluated window length using the threshold corresponding to the point the ROC diagram closest to [0, 1].
